# Patch-use dynamics by a large herbivore

**DOI:** 10.1186/s40462-015-0035-8

**Published:** 2015-03-17

**Authors:** Dana P Seidel, Mark S Boyce

**Affiliations:** Department of Biological Sciences, University of Alberta, Edmonton, Alberta T6G 2E9 Canada

**Keywords:** Home-range development, Site-fidelity, Foraging selection

## Abstract

**Background:**

An adaption of the optimal foraging theory suggests that herbivores deplete, depart, and finally return to foraging patches leaving time for regrowth [van Moorter et al., Oikos 118:641–652, 2009]. Inter-patch movement and memory of patches then produce a periodic pattern of use that may define the bounds of a home range. The objective of this work was to evaluate the underlying movements within home ranges of elk (*Cervus elaphus*) according to the predictions of this theory. Using a spatial temporal permutation scan statistic to identify foraging patches from GPS relocations of cow elk, we evaluated return patterns to foraging patches during the 2012 growing season. Subsequently, we used negative binomial regression to assess environmental characteristics that affect the frequency of returns, and thereby characterize the most successful patches.

**Results:**

We found that elk return to known patches regularly over a season, on average after 15.4 (±5.4 SD) days. Patches in less-rugged terrain, farther from roads and with high productivity were returned to most often when controlling for the time each patch was known to each elk.

**Conclusions:**

Instead of diffusion processes often used to describe animal movement, our research demonstrates that elk make directed return movements to valuable foraging sites and, as support for Van Moorter et al.’s [Oikos 118:641–652, 2009] model, we submit that these movements could be an integral part of home-range development in wild ungulates.

## Background

Home-range development and range-use dynamics are key components of foraging behaviour with implications for animal movement, habitat selection, and fitness [1,2]. The home range often is defined to be the area known by the animal and remembered or maintained because of its value, presumably in resources required by the animal for survival and reproduction [1,3,4]. However, simulations of memory processes alone have failed to yield stable home ranges [5,6] and the biological mechanisms underlying the development and maintenance of home ranges in non-territorial animals are still missing. There is a growing body of literature on mechanistic home range models hypothesizing the underlying rules for movement or landscape structure that may define or result in the development of stable home ranges [5,7-10].

Compared to traditional techniques that describe home ranges, mechanistic models are more comprehensive attempts to unveil the processes that result in home-range behaviour. Because these models are based not only on the movements of animals but upon the underlying rules for movement, they have the ability to predict an individual’s spatial use, not only describe it [9,11]. As such these models, when validated, are especially powerful tools for predicting responses to changes in habitat [9,12] either by human land-use change, or natural perturbations to the environment. Because of their potential predictive powers, numerous mechanistic home range models have been developed recently. Unfortunately, these works focus primarily on the development of defended ranges or territories of central place foragers [3,13,14], not the ranges of more diffuse foragers (e.g. most cervids) without a central place or a discrete and defended territory.

In an attempt to address this gap, a model by Van Moorter et al. [5] simulates home-range development combining the rules of optimal foraging theory and a two-part memory system. Foragers move between dynamically valued patches distributed across the landscape, removing food from a patch until depletion stimulates departure according to the marginal value theorem [15]. Their movement is biased by the utility of surrounding patches and both short-term memory and long-term memory that prevent backtracking over depleted patches while maintaining knowledge of successful patches and allowing time for forage regrowth prior to return.

Seidel and Boyce [Seidel DP, Boyce MS: Varied tastes: home range implications of foraging patch selection, forthcoming] evaluated four formative assumptions of Van Moorter et al.’s model in two populations of elk in SW Alberta. Their work formed the first empirical support for this model but they did not investigate the predicted movement patterns or returns to foraging sites. Although directed movements between areas of resource abundance where animals linger to forage have been demonstrated [16-18], few studies have shown returns or recursive movement patterns in ungulate populations and none exhibit returns directly to identified foraging patches [19,20]. As such, our objective was to evaluate movement within home ranges according to predictions of a proposed mechanistic home range model for foragers.

We used a flexible space-time permutation scan statistic (STPSS) to identify and approximate the scale of discrete elk foraging patches in space and time. We first sought to establish whether and how frequently elk return to these patches. Secondly, our goal was to identify the characteristics of a patch that increased the likelihood of reuse. Connecting patch-return likelihood to attributes of these patches and surrounding landscape lays the groundwork for understanding why and how animals use various areas within their home range and allows us to evaluate the expectation that those patches that are revisited should be of higher quality than other available patches.

## Results

### SatScan clustering

Using the STPSS procedure, 815 clusters were identified over the summer season with a total of 2,112 returns overall. Clusters with radii less than 15 m in length were removed, 47(5.8%) qualifying clusters, leaving 768 clusters for analysis. The average number of clusters identified in total each week was 54.86(±8.24 SD) clusters (minimum: 42, maximum: 63).

An average of 109.7(±8.36 SD) clusters per individual was identified over the 3-month season. The average radius of analysed clusters was 92.4 m (±39.1 SD) and included an average of 2.63(±1.21 SD) fixes in each cluster. SatScan output also provides number of observed fixes within the cluster. This value is often larger (but never smaller) than the number of fixes included in the cluster and represents the total number of fixes within the spatial boundaries of the cluster over the entire analysed temporal period, e.g. 7 days. The average number of observed fixes in each cluster was 2.77(±1.47 SD) indicating that animals frequently revisited the cluster within the same week but not within the chosen temporal window.

### Investigating returns

Our calculations suggested that across all animals, clusters were returned to an average of 2.75 (±2.37 SD) times over the 3-month season (including single fix returns). Animals returned to each cluster after an average of 15.38 days (±5.39 SD) and the average rate of return (#returns/timeknown) was 0.034 (±.027 SD) returns per day (or 3.34 returns per patch over the study period). Some clusters (17.1%) did not experience a return foraging event. See Table 1 for additional summary statistics on returns.

**Table 1:** **Summary statistics on returns for cow elk, summer 2012**

	**n**	**% unreturned**	**#UnRet (SingleRet)**	**Avg returns**	**MAXReturns**	**Avg # singles**	**Avg return rate (no singles)**
**E144**	111	20.72	23 (4)	5.22	11.00	1.10	11.96
**E146**	118	24.58	29 (6)	2.06	5.00	0.29	12.66
**E159**	117	6.84	8 (3)	3.14	7.00	0.80	20.24
**E164**	117	11.97	14 (7)	3.29	7.00	1.20	24.82
**E170**	96	15.63	15 (8)	3.21	9.00	0.83	15.54
**E172**	105	8.57	9 (4)	3.67	10.00	0.90	13.17
**E173**	104	31.73	33 (9)	2.49	7.00	0.65	9.24
**AVG**	109.71	17.15		3.30		0.82	15.38
**SD**	8.36	9.03		1.00		0.30	5.39

Across 7 cow elk, an average of 109.7 clusters per animal was detected in GPS relocations from summer 2012. An average of 17% of these clusters, presumed foraging patches, were unreturned to, however the percentage of patches unreturned to drops 2.6-8.7% when including single fix returns over the season which were not immediately considered foraging returns. The average number of returns per cluster, as well as maximum number of returns recorded, are presented for each animal and then averaged for the population. The “average return rate” is the average number of days between return events, not including singles and not accounting for differences in time known to the individual.

**Figure 1:**
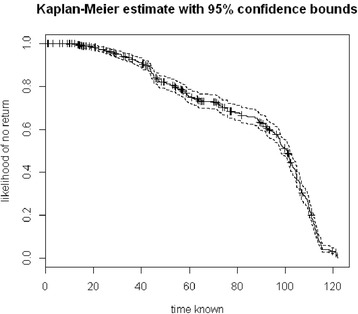
**Kaplan-Meier curve examining the influence of** ***TMKnown*** **on cluster visits**. *TmKnown*, or the number of days between an individual’s first visit to a patch and the end of the study period, has a noteworthy effect on the likelihood that an identified patch will be revisited. Revisited patches have, on average, been known for 85 days, suggesting that many clusters not returned to were potentially not known long enough to be returned to within the sampled season.

A high frequency of zeroes is often best explained by length of time that the patch was known to the elk– especially evident in Livingstone animals. For example, because E144 moved to a new area of her home range just 3 weeks before the end of the sampling period, her late-season clusters had a much shorter period of time for revisitation and account for 69.6% of her non-returned patches over only 21% of the study period. This phenomenon is explored using a Kaplan-Meier curve demonstrating that until a patch is known for about 20 days it has nearly a hundred percent chance of not being returned to but after 100 days a return is a near certainty (Figure 1).

Returns were overdispersed (mean = 2.75, variance = 5.63) and a negative binomial distribution examined for better fit. As expected, a fixed negative binomial outperformed a fixed Poisson model by 14 AIC (Akaike Information Criterion) units and reduced the Pearson χ2 dispersion coefficient from 1.34 to 1.10. When mixed-effects models were estimated with Poisson and negative binomial families, fit was improved compared to fixed models. Unexpectedly, the mixed-effects models differed only by 0.14 AIC units (mixed Poisson 2857.92, mixed NB 2857.78) but again the Pearson χ2 coefficient indicated less over dispersion with the negative binomial (1.21 to 1.13 respectively).

To explain variation in the pattern of returns, we fit biologically plausible alternative models and identified the model with the smallest AIC (see Table 2). The best fit model by AIC indicates that time known, ruggedness, distance to road and productivity at the site most significantly influenced the likelihood of return across all patches. The AIC-selected model explained approximately 13% of the deviance when compared with the null model. The dispersion parameter for the top reported model was 34.716 (25.61 SE). The width of this standard error and the magnitude of the corrective parameter were large but the parameter estimates were stable across mixed Poisson and Negative Binomial approaches and the Pearson Chi Squared dispersion parameter, 1.13, indicates that the remaining 13% overcorrelation is within suitable bounds for use of the negative binomial distribution [21].

**Table 2:** **Candidate models and akaike weights**

	**Candidate models**	**AIC**	**DeltaAIC**	**AICw**
Model M*	~ TmKnown*NDVI + Ruggedness + Herd*(DistRd), rand(ElkID)	2857.8	0.0	0.77
Model L*	~ TmKnown + Ruggedness + Herd*(DistRd) + NDVI, rand(ElkID)	2860.7	2.9	0.18
Model K*	~ TmKnown + Ruggedness + Herd*(DistRd), rand(ElkID)	2863.1	5.3	0.05
Model G*	~ TmKnown + NDVI + Herd*(DistRd + Traffic), rand(ElkID)	2878.4	20.7	0.00
**Model J**	~ TmKnown + Ruggedness, rand(ElkID)	2882.4	24.6	0.00
Model H*	~ TmKnown*NDVI, rand(ElkID)	2884.4	26.7	0.00
Model I*	~ TmKnown*Aspect, rand(ElkID)	2887.7	29.9	0.00
**Model E**	~ TmKnown + NDVI, rand(ElkID)	2888.3	30.6	0.00
**Model F**	~ TmKnown + NDVI + Aspect + Canopy, rand(ElkID)	2891.1	33.3	0.00
Model D*	~ NDVI + Herd*(DistRD + Traffic), rand(ElkID)	3180.7	323.0	0.00
**Model C**	~ NDVI + Aspect + Canopy, rand(ElkID)	3187.6	329.8	0.00
**Model A**	~ NDVI, rand(ElkID)	3193.0	335.2	0.00
**Model B**	~ NDVI + Aspect, rand(ElkID)	3195.0	337.2	0.00

*All models with interaction effects included main effect terms of interacting covariates.

The candidate model set contained 13 models comparing the influence of vegetation, physiogeographic and disturbance variables. The top model included ruggedness, TmKnown, distance to road, and productivity, receiving 77% of support in the data.

Higher relative productivity of a patch (*NDVI*) increased the likelihood of return (Table 3). Elk preferred to return to patches farther from roads and the interaction parameter between herd and distance to road was included in the top model indicating the road effect on returns was magnified in the Waterton herd. Additionally, our model shows that Waterton animals return less often than Livingston animals overall. The censorship parameter, *TmKnown*, proved to have the largest effect size, positively impacting return likelihood almost twice as much as any other variable. The longer that a patch is known by, i.e. available to, an animal the more likely it will receive a return visit. Examination of this variable using a Kaplan-Meier survival curve further emphasizes its importance in the revisitation of patches. According to our data, patches known to an elk for less than 60 days have roughly only a 25% chance of being revisited; this chance doubles once patches have been known to the elk for at least 100 days (Figure 1).

**Table 3:** **Coefficient estimates for top model, model M**

**Coefficients**	**Estimate**	**S.E.**
*(Intercept)*	0.936	0.113
*TimeKnown*	0.545	0.036
*Productivity (NDVI)*	0.093	0.032
*Ruggedness*	−0.153	0.039
*Herd.WATERTON*	−0.281	0.174
*Distance to Road*	0.051	0.035
TimeKnown*NDVI	−0.079	0.036
Herd.WATERTON*DistRd	0.104	0.051
**Random Effect: ELKID**	**Variance**	
*(Intercept)*	0.046	

## Discussion

Our results confirm that individual elk make repeated foraging visits to patches within a growing season. Furthermore, we demonstrate that distance from roads, as well as landscape ruggedness, and green herbaceous productivity contribute to increased returns at foraging patches indicating that patch value influenced the likelihood of return to a patch, just as proposed by Van Moorter et al.’s [5] home-range model.

Return behaviours have been shown before in wild ungulates, but to our knowledge, this is the first empirical demonstration of recursive movements specifically to identified foraging sites. Wolf et al. [20] and Bar-David et al. [19] both identified recursion events to previously used or “known” locations related to resources, or foraging behaviours, though neither estimated returns directly to identified foraging areas. By analysing return patterns to a specific location and use, we uniquely explored how foraging selection might drive movement patterns.

**Figure 2:**
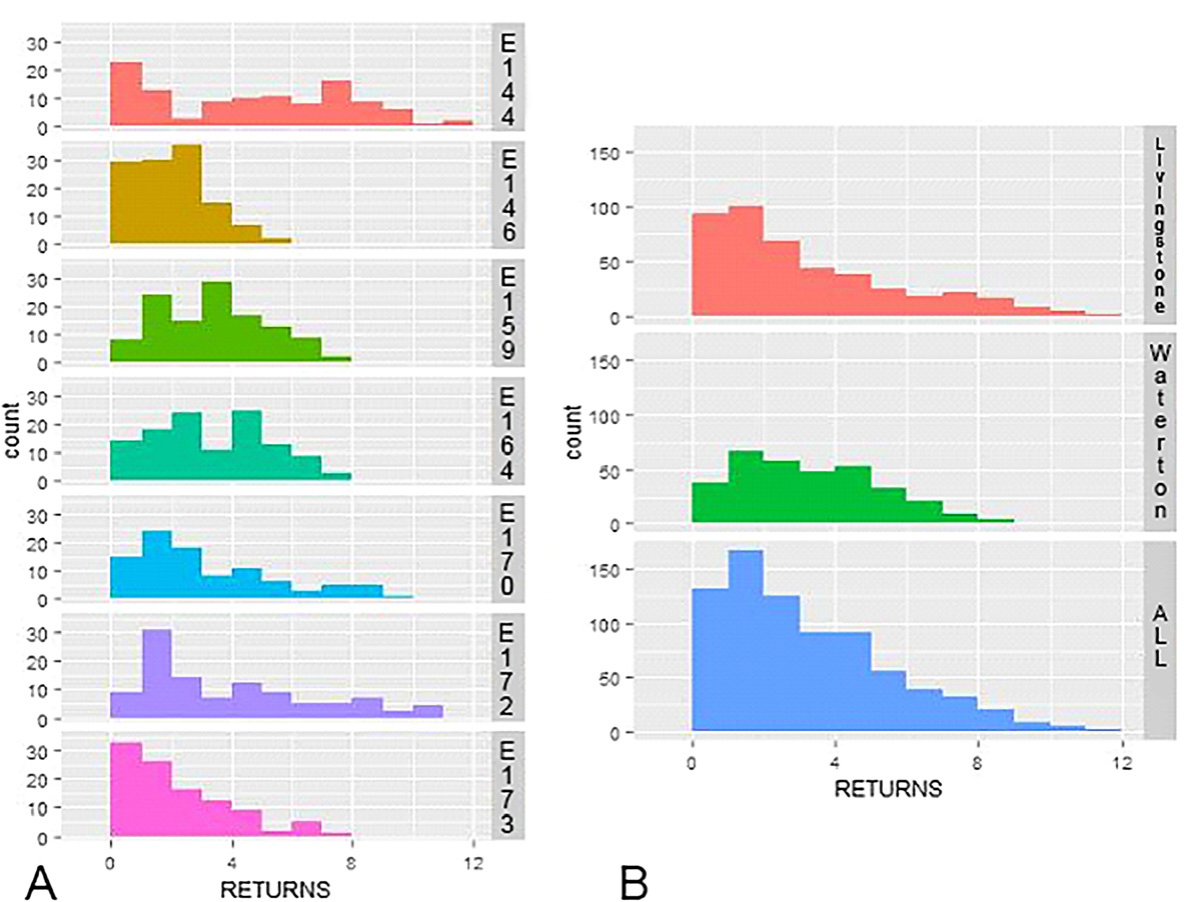
**Distribution of return frequency to clusters by (A) Individual and (B) Herd across the summer season.** Histograms depicting frequency of returns to identified foraging patches are presented for each individual cow and each herd cumulatively. These histograms demonstrate the wide variation present across individual and herd return frequencies, potentially influenced both by differences in habitat and behaviour across the season.

Differences across return distributions of individuals and across herds were noted (Figure 2A&B), with Waterton animals returning less often overall. These distributions are likely influenced by subtle range shifts over the season and by individual movement behaviours. Larger home ranges lead to fewer returns and longer time between returns at individual patches [van Moorter B: unpublished manuscript]. This is logical: when there is more space to cover and more patches to visit, the time between returns will be longer leading to fewer returns over a single season. Movement between (and thus return rates to) patches could be influenced by other environmental features such as ruggedness of terrain or overall extent of home range although we did not explore these explicitly in this analysis. We observed that Waterton cows expanded their home ranges over the course of the season, but maintained returns to the entire area, even as it expanded late in the summer down into the aspen forests and wetlands on the east shore of lower Waterton lakes where bull elk typically concentrated their summer movements. Maintenance of larger home ranges may explain a portion of the reduced return likelihood of Waterton patches.

Our top model demonstrates that at the population level, *TmKnown*, ruggedness, productivity, distance to road, and interactions between distance to road and herd and *TmKnown* and productivity were the most influential environmental covariates determining return counts at patches across the season. The importance of productivity in return models supports the underlying thesis of Van Moorter et al.’s [5] model which values patches based on replenishment of resources. As expected our results demonstrate that productive patches are returned to more often than less productive patches. An attraction to productive forage is consistent with previous work demonstrating that elk migration often follows the start of spring photosynthetic activity, or greenup; as new growth extends into higher elevations over summer so do elk [22]. Forage research on elk also shows attraction to intermediate levels of biomass, often more digestible and productive than tall late-season stands, and forage abundance has been shown to encourage site fidelity in nonmigratory elk populations on short time intervals, supporting our results that productivity may strongly influence returns [23-25].

Distance to nearest road and its interaction with the Herd variable appeared in the top model, with Waterton animals being more sensitive to road proximity. Animals in national parks often seem undisturbed by roads, habituated to traffic and people, and attracted by the roadside vegetation and protection from predators that roads and human settlements offer [26], but in other populations, especially in those facing hunting pressure, roads and high traffic have been shown to alter movement near roads [27,28]. From the perspective of foraging, human disturbance has been shown to increase vigilance, reducing time spent foraging, foraging efficiency, and intake [29-31] and, recently, to deter foraging patch selection in elk [Seidel DP, Boyce MS: Varied tastes: home range implications of foraging patch selection, forthcoming]. Our analysis demonstrates that disturbance also might affect whether or not that animal returns to patches over time.

Inclusion of the *TmKnown* variable markedly improved the fit of our model to the data and emphasizes the temporal dynamics at play driving returns. *TmKnown* was the strongest indicator of return likelihood, with an effect size nearly twice that of any other predictor; this is a logical result. Patches visited earlier in the season have a longer period of time during which they can returned. The Kaplan-Meier estimation demonstrates clearly that patches must be known for roughly 20 days before attracting a return (Figure 1). Given the time needed for regrowth, revisits before 20 days would likely be disadvantageous giving further support to the Van Moorter et al. model [5]. Additionally this figure demonstrates that nearly all patches known for at least 115 days were revisited and displays a sharp uptake in revisits once a patch was known for 90 days or more. Exhibition of return behaviour overall indicates that animals are not avoiding previous locations and that previous use may increase subsequent use, just as demonstrated by Wolf et al. [20]. If this coefficient had been diminished or even negative, we would expect that animals were likely moving into novel environments, not cycling back over the season either due to range drift or possibly resource depletion or predator avoidance. In future research, it would be useful to explicitly evaluate how the demonstrated increase in return probability over time compares to probabilities extracted from simple biased random walk models (i.e. biased to a central location, considering both mono- and multi-nuclear models), or more advanced multi-phasic movement models. Such a comparison of models, using empirical data for parameterization, could be very informative and offer a unique evaluation of current proposed models for understanding movement and space use of large mammals.

Traditionally, simple random-walk or diffusion models have been used widely to model animal movement and, dependent on the time scale in question, can provide a realistic approximation of movement for many species [32]. Diffusion alone however does not result in emergent home-range behaviour; using a diffusion approach, eventually the paths of an animal will expand to fill any available extent. Diffusion models with an attraction vector to a central place (e.g. a den, a nest) can result in a circular, unimodal, home ranges but empirical observation shows that animals’ real home ranges generally exhibit multimodal use with non-circular edges [32]. Mechanistic home range models have evolved in an attempt to identify and model the movement processes that can simulate emergent multimodal utilization distributions and realistic home-range boundaries (see [3,32] for further review of recent movement and home-range modelling). The Van Moorter et al. [5] model, predicting a foraging and memory-driven model, provides a realistic model for the intra-home-range movement in wild ungulates, without requiring presupposition of home range centers or a single attractive nuclei. Our field observations have demonstrated repeated movements among multiple nodes of attraction which are indicative of memory processes, and negate simple diffusion or central place models for ungulate home range development.

## Conclusions

We have demonstrated that elk will return to foraging patches repeatedly over the season. Return behaviour should be driven in part by patch value, and indeed, we show that productivity, terrain ruggedness, and proximity to road all influenced the likelihood that elk would return to foraging patches. These results demonstrate that the Van Moorter et al. [5] model for home-range development appropriately characterizes key aspects of elk foraging and movement behaviour and furthers understanding of within home range movement of free ranging elk. Increased research into the mechanisms driving space use and empirical evaluation of theoretical home range models will improve our understanding of the dynamic nature of animal space use and movements, especially in response to human land-use change.

## Methods

### Study area & animals

Elk in this study ranged freely within the montane ecosystem of SW Alberta. The study area is characterized by steep mountainous terrain to the west, abruptly transitioning in the east to rolling grasslands and agricultural land. Seven cow elk from two herds (Waterton and Livingston) were included in these analyses. The three Waterton animals ranged within the boundaries of Waterton Lakes National Park, and were predominately associated with the Park’s northwestern hills and the aspen forests and wetlands southeast of Lower Waterton Lake. Tourism to the national park during summer is a unique disturbance for animals in this herd. The four radiocollared Livingstone animals ranged on both sides of the Livingstone Range, an eastern ridge of the Rocky Mountains where they encountered timber cut blocks of varying age and dense forests dominated by lodgepole pine (*Pinus contorta*) to the west, and rolling agricultural and range lands to the east.

### Clustering

To identify patches used for foraging, a retrospective space–time permutation scan statistic (STPSS) was used to identify clusters in the relocation data for each individual elk using SaTScan® [33]. The scan statistic is defined by a moving cylindrical window with a base in geographic space and height defined by time. Using this method, each relocation was considered to be the center of a possible cluster (containing a minimum of 2 fixes) across multiple spatial windows and at each available time window (i.e., over 1 day, 2 days, or 3 days). The analysis considers all relocations within a wide range of cylinders when evaluating for clusters: everything from relocations within tall poles, i.e. small spatial windows but across many days, to those that might be described to occur within wide flat discs, i.e. large spatial windows during a single day [34]. For detailed information on the probability function underlying this clustering method, see Kulldorff et al. [33].

Following adaptations explained by Webb et al. [34] to use this method with GPS relocation data, we let *c*_*zd*_ = number of locations at geographic coordinate *z* during day *d*, and defined *C*, the total number of observed GPS elk locations, as

$$ C={\displaystyle \sum_z{\displaystyle \sum_d{c}_{zd}}} $$

On day *d* at location *z* the expected number of GPS locations (*U*) is

$$ {U}_{zd}=\frac{1}{C}\left({\displaystyle \sum_z{c}_{zd}}\right)\left({\displaystyle \sum_d{c}_{zd}}\right) $$

Because each relocation point in a GPS dataset is unique, the number of GPS locations at a location *z* across all days sums to one and, subsequently, *U*_*zd*_ =1. Expected number of locations *U*_*A*_ in a cylinder *A* is the summation of these expectations within that cylinder:

$$ {U}_A={\displaystyle \sum_{\left(z,d\right)\in A}{U}_{zd}} $$

When there is no space–time interaction, *c*_*A*_, the observed number of locations within the cylinder, is distributed according to a hypergeometric distribution with mean *U*_*A*_ and probability function:

$$ P\left({c}_A\right)=\frac{\left(\frac{{\displaystyle {\sum}_{z\in A}{c}_{zd}}}{c_A}\right)\left(N-\frac{{\displaystyle {\sum}_{z\in A}{c}_{zd}}}{{\displaystyle {\sum}_{d\in A}{c}_{zd}-{c}_A}}\right)}{\left(\frac{C}{{\displaystyle {\sum}_{d\in A}{c}_{zd}}}\right)} $$

When both the number of geographic locations and the number of days within a cylinder are small compared to *C*, *c*_*A*_ is expected to be approximately Poisson distributed with mean and variance *U*_*A*_. As such, the evidence that a given cylinder contains a cluster can be measured by a Poisson Generalized Likelihood Ratio.

Elk most actively forage during crepuscular periods [35-37] thus, to help ensure that clustering could identify patches primarily used for foraging and not some other activity, e.g., grooming or bedding, data from peak hours of day and night were removed (10:00–14:00 and 22:00–2:00) prior to clustering. In addition, all resulting clusters with a radius ≤ 15 m were removed because these likely represent GPS error on resting or bedded animals [23]. Three decision rules had to be made prior to running the scan statistic: the maximum spatial window, the maximum temporal window, and permission for geographic overlap of clusters.

Frair et al. [23] used a first-passage time analysis, assessing how long an animal spends in an area of a given size, to identify the scales at which three separate movement processes occurred: resting, foraging, and traveling from 2-hr fix data. When foraging, female elk travelled an average of 265.7 m (42.5 m SD) between fixes; accounting for this previous work and given the logistical constraints of our field sampling, a maximum diameter of 300 m was chosen as an upper spatial bound for analysis. The maximum number of sequential days evaluated for clusters of points, i.e., the maximum temporal window, was left broad: including up to 3 days of points. Finally, within an individual scan (over the data of one elk for a single week), no geographic overlap was allowed between reported clusters; this is a constraint imposed to ensure that we captured unique patches in space.

### Counting returns

After identifying the boundaries of foraging patches, we recorded all revisits by an elk to its known patches during the summer season. Patches were identified weekly from telemetry data for each animal and were aggregated from June-August 2012 for return analyses. Returns to each patch were calculated for the entire duration of the summer season. Sampling began the first week of June to reduce the likelihood of including patches encountered on spring migration to the summer range as these patches are unlikely to be used again within the season.

For purposes of our analysis, a return was defined to be a series of 2 or more sequential fixes within 300 m of the cluster point separated by more than 3 days (i.e., 36 fixes) from the previous visit. This mirrored the spatial rule used for defining clusters by the STPSS (maximum 300 m diameter) and required a temporal window that would help to ensure that animals left the general area and subsequently returned in a separate event. Elk often spend several days encamped in one area and then relocate to another distant area of their home range [17]; we expected these rapid relocation events to occur within our 3-day buffer and to separate one series of cluster visits from another. Single fix events within the appropriate spatial and temporal definition were denoted as “singles” but were not assumed to represent a foraging event. Biologically, we hypothesize these single fix events could represent exploratory returns to assess biomass regeneration in the presence of competing herbivores (e.g., cattle) but given their duration were not considered to be a foraging return for this analysis.

**Figure 3:**
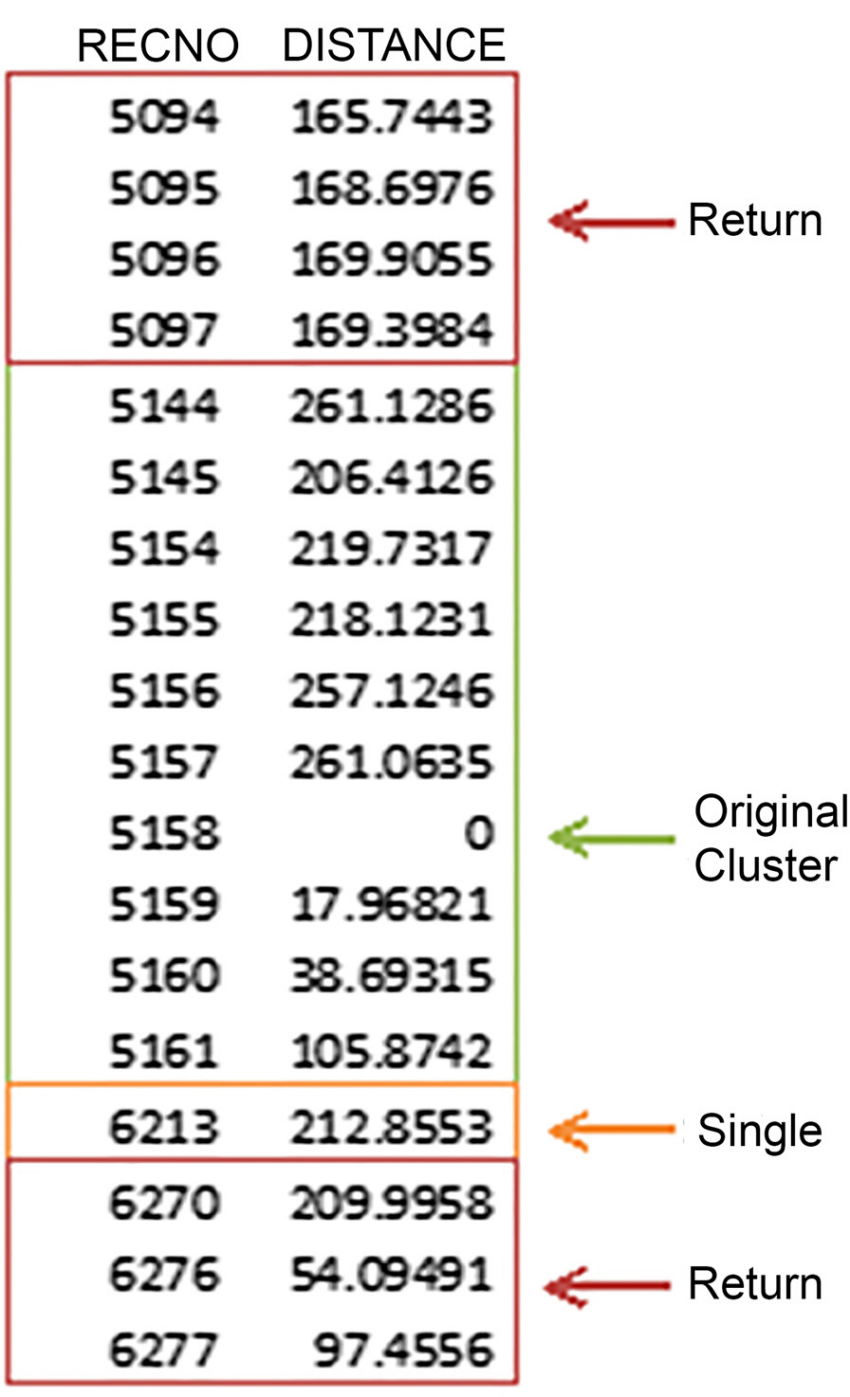
**Example subset table for differentiating return events.** This example patch has received 2 returns and 1 single fix event over the season. Note that a return can occur prior to the event clustered by the space-time permutation scan statistic.

To count returns to each patch, we first imposed the spatial boundary of the patch and then tallied return events. Distances between each relocation for an animal and each cluster for that animal over the study period were calculated using Geospatial Modelling Environment (GME) [38]. In Program R [39], we identified the subset of fixes within 300 m of a cluster point. This subset contained all returns to the 300 m buffer including the foraging event originally clustered, but at this point they are undifferentiated events (See Figure 3). To accurately count the number of returns to a site, we used the sequential fix numbers (adjusted for missing fixes) included in the subset table to isolate clusters in time. Using the diff function in GME, the table was read separating events of sequential fixes. In this way, nonsequential points outside the 3-day buffer represented start points of events that were isolated and tallied, separating single-fix events from multi-fix events, or returns. Based on this method, the number of returns to an area equals the (number of events in the area) – 1, accounting for the originally clustered foraging event. A correction to the returns count was needed in instances when the final record was a single-fix return: in this case, returns equal (number of events in the area) – 2, accounting for both the last single event and the original cluster point.

### Return analysis

Using counts of returns to a patch as our response variable, we sought to model how environmental covariates might influence an elk’s decision to return to patches later in the season using an information-theoretic approach for model selection [40]. All covariates were standardized to mean = 0 and SD = 1, and using mixed negative binomial regression through the glmmADMB package in Program R [39], we investigated which environmental covariates influenced the incidence of return count data at 768 clusters.

Our model set included 13 biologically relevant candidate models to explore the influence of environmental and anthropogenic factors on the number of times a patch was revisited (Table 2). Ungulates move to maximize forage intake and typically seek out areas of intermediate biomass with highest quality and quantity of available forage plants [41]. As such, productivity and vegetation models were included to explain the variation in the number of returns to a patch.

**Figure 4:**
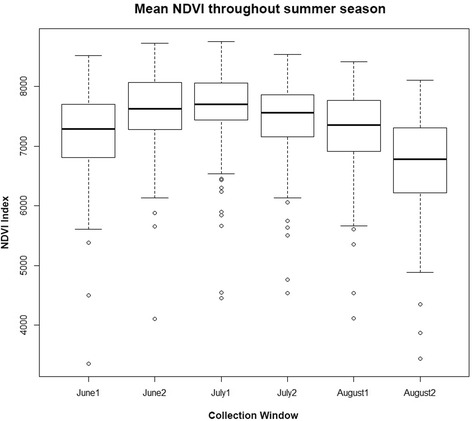
**Boxplot demonstrating mean NDVI and its variance throughout summer.** MODIS satellites retrieve imagery from the study site every 16 days, twice each month. The 6 boxplots present the average and variance of Normalized Difference Vegetation Index (NDVI) values for each photoperiod. These averages and variances are calculated from NDVI values reported at all clusters identified. The first reporting period of July (July1) has the highest mean and the lowest variance making it the best choice for a parameter demonstrating relative productivity of each cluster. The higher variance early and late in the season is likely due to timing variation of snow melt, growth, and die-off along elevation and cover gradients all of which influence NDVI values.

Model A tests the idea that returns are solely related to relative productivity of the patch. Higher productivity is expected to shorten regrowth times and provide more available biomass over the season, potentially increasing the number of returns occurring over the time window by decreasing the number of days between returns. The normalized difference vegetation index, NDVI, an index of above-ground primary productivity, was compiled from images collected by MODIS remote-sensing satellites during May through September 2012 at a 250 m resolution every 16 days. The mean NDVI value of all clusters in each reporting period demonstrates the typical parabolic trend in productivity values over the summer (see Figure 4). Extracting the NDVI value at each cluster during peak productivity (early July) allowed us to include a covariate indicating the relative productivity of each patch during the summer season. Elk respond to physiogeographic features that determine where forage is most available (e.g. ruggedness, slope, elevation, aspect). Differences in elevation, slope, and aspect can create microclimates that affect localized productivity and available forage [23] and subsequently may affect elk movement [42].

As a secondary variable influencing productivity, northness, or cos(*Aspect*), was used for interpretation of the circular variable aspect in models. Model B includes this second productivity related parameter, *Aspect,* to assess how hillshade may play a role in return likelihood in addition to relative productivity (NDVI). In addition to seeking out forage, research has shown that elk movement can be driven by predator avoidance [23,42]. Remaining close to or within cover is an important predator avoidance strategy for elk [29]. To evaluate the influence of cover on return frequency, *CanopyClosure* was extracted from a 2005 map created by the Foothills Research Institute [43]. This cover map is a composite of remotely sensed LandSat data with 30-m resolution on land cover and crown closure, as well as species composition, and agricultural and regeneration masks. Model C includes *Canopy, Aspect, NDVI*, for a full vegetation model, accounting for the importance of cover for predator avoidance [29], and the attraction of productive forage [41].

Human disturbance from road networks potentially acts as a deterrent to returning elk. The road network described in a traffic model developed for our study area [44] was used to obtain estimates for the distance to road, *DistRd*, and average summer daily traffic on nearest road, *Traffic*. In Waterton National Park, high levels of tourist traffic push through the park’s few roads daily. In Livingstone, the landscape contains small, seldom-travelled roads. Due to the large difference in road density and traffic between the two herds, a binary and categorical covariate of *Herd* was included in models and allowed to interact with *Traffic* and *DistRd* variables. The *Herd* variable specifies whether a patch occurs within the boundaries of the Livingstone or Waterton herds and was included to account for differences in the impact of roads and traffic on return likelihood to patches across the two herds. Model D incorporates the effect of these human disturbances as well as the baseline productivity of a patch (NDVI) that we hypothesize attracted returns.

The *TmKnown* covariate refers to the number of days elapsed between the first ever visit to the patch by an elk and the end of the sampling period at the end of August. This variable accounts for the increased likelihood of revisitation that some patches have over others in the dataset just based on when they were first encountered in the season and the length of our sampling period. Additionally, this covariate has some simple biological relevance accounting for animal learning and memory. The longer a patch is known to an animal, and the longer we monitored returns to it, the more returns that patch is likely to accrue. Model E added *TmKnown* to the baseline productivity model (Model A). Similarly, Model F added *TmKnown* to the complete vegetation model (Model C) and Model G considers *TmKnown* within the productivity and disturbance model (Model D). These function as direct comparisons for the effect of *TmKnown* on return likelihood. Interactions between *TmKnown* and productivity parameters (*NDVI* and *Aspect*) were included to test for the potential temporal variation in the attraction of patches; it is possible that patches might be returned to more or less over the time they are known based on their productivity across the summer (Model H and I).

Movement by elk is restricted by rugged terrain and we hypothesize that the returns would be more frequent at less-rugged patches because they likely require less energy for travel to and within [29,44,45]. Terrain ruggedness, *Ruggedness,* was included in models to reflect this predicted influence on movement [29]. Model J includes just *Ruggedness* and *TmKnown*, representing the hypothesis that returns are only explained by the accessibility of the patch and how long it has been known. In a model representing landscape terrain, Model K includes both road networks and the *Ruggedness* of the terrain as well as the *TmKnown* variable*.* Model L and Model M represent combinations of the terrain model with the productivity parameter (NDVI) and its interaction with *TmKnown*.

Individual variation in return patterns was substantial (see Figure 2A) and *ElkID* was included as a random effect in all candidate models to account for this variation. Although differences in terrain ruggedness were visually identifiable across herds, an interaction between ruggedness and herd was not expected to influence return frequency. That is to say, the return behaviour of Livingstone animals was not influenced differently by ruggedness than was the behaviour of Waterton animals, despite the greater overall ruggedness of Livingstone terrain. All of the observed differences between herds were attributable to the difference in tourism levels between areas and individual variation accounted for by the random effect. Finally, the influence of the *TmKnown* parameter on return likelihood was examined using a Kaplan-Meier survival curve built using the survival package in Program R [39].
